# Ventral STN stimulation is associated with improved axial motor outcomes in Parkinson’s disease

**DOI:** 10.1007/s00702-025-02931-1

**Published:** 2025-04-24

**Authors:** Madison Butler, Asra Askari, Brandon Zhu, Kara Wyant, Daniel Leventhal, Parag G. Patil, Kelvin L. Chou

**Affiliations:** 1 University of Michigan, Neurology, Ann Arbor, MI 48105 USA; 2 University of Michigan, Neurosurgery, Ann Arbor, USA; 3 Biomedical Engineering, University of Michigan, Ann Arbor, USA

**Keywords:** Axial motor signs, Subthalamic nucleus, Parkinson’s disease, Deep brain stimulation

## Abstract

Subthalamic nucleus (STN) deep brain stimulation (DBS) is well-established for improving appendicular motor signs but its effect on axial motor signs is less clear. Additionally, the location of active electrode contact within the STN has been shown to differentially affect motor outcomes. We investigate the effect of STN DBS and the role of active electrode contact location on axial motor outcomes. Axial scores were assessed in 70 patients with advanced PD between 6 and 12 months after bilateral STN DBS. Repeated measures one-way ANOVA was performed to compare the mean axial motor scores between different medication and stimulation treatment conditions. Multiple linear regression was performed to determine the association between electrode contact location and axial motor score. The mean duration of follow-up was 7.37 ± 2.49 months. The mean total axial score was improved with STN DBS compared to the OFF MED-OFF STIM condition (6.36 ± 4.50 vs. 8.91 ± 5.49, *p* < 0.0001). A more dorsal electrode contact location on the right was associated with increased (worsened) total axial score (slope = 0.407, *p* = 0.0047). Total axial score was also lower (improved) in the anterior ventral STN region but not the posterior ventral STN region on the right. STN DBS improves total axial score and several axial subscores in patients with PD 6–12 months postoperatively. A more ventral electrode contact location on the right was associated with improved axial score. Additional research is warranted to determine if this is the optimal stimulation location to improve axial signs in other DBS cohorts.

**Supplementary Information:**

The online version contains supplementary material available at 10.1007/s00702-025-02931-1.

## Introduction

Parkinson’s disease (PD) is the second most common neurodegenerative disorder (Homayoun 2018). Clinical manifestations of the disease include resting limb tremor, rigidity, and bradykinesia, which respond well to dopaminergic treatment. Axial symptoms, such as impaired gait, postural instability, and impaired speech, frequently present as the disease progresses and are less responsive to dopaminergic therapy compared with appendicular symptoms (Fasano et al. 2015).

Deep brain stimulation (DBS) has emerged as an effective therapeutic option for patients with advanced PD, particularly for patients with complications such as dyskinesias and wearing off (Ramirez-Zamora and Ostrem 2018). However, there is variation in the amount of symptom improvement between patients, which may be due to the location of stimulation within the STN. We previously demonstrated that the loci of maximal motor improvement in PD after DBS (dorsal and posterior to the STN midpoint) was distinct from the loci for maximal dysarthria (ventral and anterior to the STN midpoint) (Kluin et al. 2022). Stimulation of the dorsolateral (sensorimotor) STN has been shown to be associated with better motor outcomes, while non-motor outcomes such as neuropsychiatric symptoms are associated with the anterior ventral associative/limbic STN subregions (Guo et al. 2021; Zhang et al. 2021; Petry-Schmelzer et al. 2019; Zhang et al. 2022). The role of STN electrode location on axial outcomes is less well understood.

Axial symptoms in PD patients have significant clinical impact as they contribute to decreased mobility and recurrent falls, and axial features of PD are associated with poorer quality of life in patients (Fasano et al. 2015; Schrag et al. 2000; Perez-Lloret et al. 2014). Moreover, the severity of axial impairment was shown to be a predictive factor of mortality after DBS in a long-term study of 143 patients with PD (Lau et al. 2019). Further research of the effect of DBS on axial symptoms to improve patient outcomes is warranted. Here, we investigate the effect of bilateral STN DBS on axial signs and the role of electrode contact location on axial motor scores. We hypothesize that STN DBS improves axial motor score in PD patients and the location of stimulation within the STN may differentially impact axial motor improvement.

## Methods

### Patient selection

Patient data was extracted from the University of Michigan Surgical Therapies Improving Movement program database, consisting of patients with advanced PD treated with STN DBS at the University of Michigan. Patients were selected for STN DBS as previously described (Chou et al. 2013). Patients were not eligible if they had dementia or structural abnormalities on brain MRI that interfere with accurate lead placement, such as tumors or severe hydrocephalus. All patients provided written informed consent, and the study was approved by the Medical Institutional Review Board of the University of Michigan.

From the extracted data, we included patients that underwent bilateral stimulation with complete electrode contact location data and motor scores for all treatment conditions including Baseline OFF MED, Baseline ON MED, off medication + off stimulation (OFF MED-OFF STIM), on medication + off stimulation (ON MED-OFF STIM), off medication + on stimulation (OFF MED-ON STIM), and on medication + on stimulation (ON MED-ON STIM). Those who were able to completely come off of medications at first follow-up were also included in the analysis using their OFF medication scores as their ON medication scores. Only patients with this data for their baseline and first follow-up visit between 6 and 12 months were included. One patient was later excluded due to a missing data point in one of the treatment conditions. The final number of patients included in the study was 70. Disease duration was calculated by age at baseline minus age at diagnosis. LED reduction was calculated by baseline (pre-surgery) LED minus post-surgery LED.

### Surgical procedure and imaging of the STN

The surgical procedure was performed as described previously (Conrad et al. 2018). Briefly, all patients were implanted with two quadripolar DBS electrodes (Model 3389, Medtronic Inc., Minneapolis, MN) with Medtronic implantable pulse generators. Patients underwent a stage I procedure in which the STN was identified electrophysiologically by microelectrode recordings, and DBS leads were inserted under fluoroscopic visualization and tested intraoperatively. Patients underwent placement of the pulse generator and connecting wires in the stage II procedure. The stage I and II procedures were performed 2–4 weeks apart. The STN was visualized using the validated imaging protocol previously described (Patil et al. 2012). The point midway between the STN rostral and caudal poles on 3T MRI was defined as the STN midpoint (Houshmand et al. 2014). Initial DBS programming was performed approximately 2–3 weeks after the second stage.

### Electrode contact localization

Postoperative CT scans were performed to visualize electrode leads 2–4 weeks after the stage I procedure. The postoperative CT was co-registered to the preoperative 3-T MRI (Analyze, AnalyzeDirect Inc.). The electrode contact localization was performed only for active contacts. MATLAB (Mathworks, Inc., Natick, MA, USA) was used to calculate the active contact position based on the best fit of the distance along the line from the DBS lead as it entered and exited the MR-visualized STN (Conrad et al. 2018). If there was more than one active contact, the average of their center coordinates was used as the active contact location. The locations of the electrode contacts were each defined as three coordinates along the x-axis (lateral-medial), y-axis (anterior-posterior), and z-axis (dorsal-ventral), relative to the STN midpoint in Talairach space. The lateral, anterior, and dorsal directions relative to the intercommissural plane were defined as positive.

### Assessment of motor scores

Motor scores were assessed according to part 3 of the Movement Disorder Society Unified Parkinson’s Disease Rating Scale (MDS-UPDRS). In this study, we included total motor score, total axial score, and axial subscores including speech (3.1), neck rigidity (3.3a), arising from chair (3.9), gait (3.10), freezing of gait (3.11), postural stability (3.12) and posture (3.13). Total axial score was defined by the sum of these axial subscores which range from 0 (normal) to 4 (severe). Total motor score was defined by the sum of all parts in part 3 of the MDS-UPDRS.

Pre-surgery baseline data was collected when patients were off medication (Baseline OFF MED) and on medication (Baseline ON MED). Post-surgery data was collected at the follow-up visit for four treatment settings– OFF MED-OFF STIM, ON MED-OFF STIM, OFF MED-ON STIM, and ON MED-ON STIM. Medications were withheld for 12 h (8 pm to 8 am) prior to assessment of medication OFF conditions, and scores in the ON STIM and OFF STIM conditions were recorded 1 h after stimulation was turned on or off, respectively.

### Statistical analysis

Repeated measures one-way ANOVA with the Geisser-Greenhouse correction was performed to compare the mean motor scores between the six treatment conditions: Baseline OFF MED, Baseline ON MED, OFF MED-ON STIM, ON MED-OFF STIM, OFF MED-ON STIM, and ON MED-ON STIM. Turkey multiple comparison test was used to correct for multiple post-hoc comparisons.

Least squares multiple linear regression was performed to investigate the quantitative effect of electrode contact location on total axial score (OFF MED-ON STIM), controlling for reduction in LED, improvement in total motor score, and total axial score (OFF MED-OFF STIM). Improvement in total motor score was calculated by subtracting the total motor score in the OFF MED-ON STIM setting from the total motor score in the OFF MED-OFF STIM condition. Hierarchical cluster analysis was performed using Ward’s method (interval: squared Euclidean distance) separately for right and left coordinate data. The agglomeration schedule was used to identify the optimal number of clusters. Based on the level at which the agglomeration coefficients began to increase markedly, the optimal number of clusters was 5 on the right and 4 on the left (Online Resource 1). K-means cluster analysis was then performed separately for right (*k* = 5) and left (*k* = 4) coordinate data. Least squares multiple linear regression was then performed to investigate the effect of cluster location on total axial score (OFF MED-ON STIM), controlling for reduction in LED, improvement in total motor score, and total axial score (OFF MED-OFF STIM). This clustering model allows us to depict the location of the electrode contact on the traced STN which helps with surgical targeting of lead placement within STN subregions.

Descriptive analyses, repeated measures one-way ANOVA, and multiple linear regression analyses were performed using GraphPad Prism Version 10.4.1 (532). Hierarchical and K-means cluster analysis was performed using SPSS Version: 30.0.0.0 (172). P values < 0.05 were considered statistically significant.

## Results

### Patient characteristics

There were 70 total patients included in the analysis; patient characteristics are shown in Table 1. Forty-nine (70%) of the patients were male and 21 (30%) were female. There were 62 (88.6%) right-handed patients and eight (11.4%) left-handed patients. The average age at diagnosis was 52.2 ± 8.0 years, and the average disease duration was 10.5 ± 4.7 years. The average baseline (pre-surgery) LED was 1528 ± 631 mg and average reduction in LED was 772 ± 534 mg. Baseline total motor score (off medication) was 42.9 ± 15.4, and baseline total axial score (off medication) was 8.64 ± 4.93. The mean duration of follow-up was 7.37 ± 2.49 months.

**Table 1:** Patient characteristics (*N* = 70)

		*N* (%)
*Gender*	*Male*	49 (70)
	*Female*	21 (30)
*Handedness*	*Right*	62 (88.6)
	*Left*	8 (11.4)
		*Mean ± SD*
*Age at diagnosis* *(years)*		52.2 ± 8.0
*Age at baseline* *(years)*		62.7 ± 7.4
*Disease duration* *(years)*		10.5 ± 4.7
*LED at baseline* *(mg)*		1528 ± 631
*LED at first follow up visit* *(mg)*		756 ± 493
*Reduction in LED* *(mg)*		772 ± 534
*Baseline axial score OFF med*		8.64 ± 4.93
*Baseline total motor score OFF med*		42.9 ± 15.4
*Mean duration of follow-up* *(months)*		7.37 ± 2.49
*Voltage (V)*	*Left*	2.51 ± 0.72
	*Right*	2.59 ± 0.68
*Pulse width (µs)*	*Left*	60.4 ± 3.38
	*Right*	61.2 ± 5.84
*Frequency (Hz)*	*Left*	138 ± 22.2
	*Right*	139 ± 22.4

### Effect of STN DBS on axial motor scores

Repeated measures one-way ANOVA was performed to compare motor scores between the six treatment conditions, including pre-surgery Baseline OFF MED, Baseline ON MED, and post-surgery treatment conditions OFF MED-OFF STIM, ON MED-OFF STIM, OFF MED-ON STIM, and ON MED-ON STIM (Table 2). At baseline, total motor score, total axial score, and all axial subscores were decreased in the Baseline ON MED condition compared with Baseline OFF MED, suggesting dopaminergic responsiveness (all p values ≤ 0.0051). To assess the effect of stimulation most directly, we focused on the difference between motor scores in the OFF MED-ON STIM condition vs. OFF MED-OFF STIM at follow-up rather than comparing to the pre-surgery baseline scores.

**Table 2:** Mean MDS-UPDRS part III scores in each treatment condition (mean ± SD), compared using repeated measures one-way ANOVA

	Baseline OFF MED	Baseline ON MED	OFF MED-OFF STIM	ON MED-OFF STIM	OFF MED-ON STIM	ON MED-ON STIM
Total motor score	42.9 ± 15.4	20.5 ± 12.0 *	47.4 ± 16.1 †	34.2 ± 14.7 *†‡	27.7 ± 13.0 *†‡§	18.8 ± 10.3 *‡§
Total axial score	8.64 ± 4.93	4.36 ± 3.85 *	8.91 ± 5.49 †	6.54 ± 4.61 *†‡	6.36 ± 4.50 *†‡	4.87 ± 3.89 *‡§
Speech (3.1)	1.06 ± 0.700	0.771 ± 0.685 *	1.26 ± 0.774 †	1.03 ± 0.613 ‡	1.14 ± 0.822 †	1.19 ± 0.804 †
Neck rigidity (3.3a)	1.63 ± 1.17	1.04 ± 1.03 *	1.79 ± 1.30 †	1.40 ± 1.18 ‡	1.07 ± 1.16 *‡§	0.700 ± 0.874 *‡§
Arising from chair (3.9)	0.886 ± 0.941	0.200 ± 0.628 *	1.09 ± 1.23 †	0.686 ± 1.08 †‡	0.714 ± 0.950 †‡	0.371 ± 0.871 *‡§
Gait (3.10)	1.70 ± 1.04	0.771 ± 0.951 *	1.73 ± 0.977 †	1.31 ± 0.894 *†‡	1.29 ± 0.980 *†‡	1.03 ± 0.884 *‡§
Freezing of gait (3.11)	0.586 ± 1.19	0.114 ± 0.553 *	0.586 ± 1.11 †	0.286 ± 0.887 ‡	0.214 ± 0.720 *‡	0.114 ± 0.553 *‡
Postural stability (3.12)	1.39 ± 1.30	0.557 ± 1.07 *	1.50 ± 1.47 †	1.03 ± 1.31 †‡	1.11 ± 1.36 †‡	0.800 ± 1.24 *‡
Posture (3.13)	1.40 ± 1.15	0.900 ± 0.980 *	0.971 ± 1.02 *	0.800 ± 0.844 *‡	0.814 ± 0.873 *‡	0.671 ± 0.794 *‡

* significant compared with Baseline OFF MED

† significant compared with Baseline ON MED

‡ significant compared with OFF MED−OFF STIM

§ significant compared with ON MED−OFF STIM

The mean total motor score and mean total axial score were decreased in the OFF MED-ON STIM condition compared to OFF MED-OFF STIM (both *p* < 0.0001). The following mean axial subscores were decreased in the OFF MED-ON STIM setting compared to OFF MED-OFF STIM: neck rigidity (*p* < 0.0001), arising from chair (*p* = 0.0041), gait (*p* < 0.0001), freezing of gait (*p* = 0.0031), postural stability (*p* = 0.0220) and posture (*p* = 0.0079). There was no difference for speech.

Mean motor scores were further improved in the ON MED-ON STIM setting compared with the ON MED-OFF STIM setting in the following: total motor score (*p* < 0.0001), total axial score (*p* < 0.0001), neck rigidity (*p* < 0.0001), arising from chair (*p* = 0.0018), and gait (*p* = 0.0002). Motor scores for speech, freezing of gait, postural stability, and posture were not further improved with the addition of stimulation to the medication ON setting.

Overall, our data suggest that STN DBS results in improved total motor score, total axial score, and several axial subscores including neck rigidity, arising from chair, gait, freezing of gait, postural stability, and posture, but not speech. There is an additional benefit of combined stimulation plus medication compared with medication only for improving total motor score, total axial score, neck rigidity, arising from chair, and gait.

### Effect of electrode coordinates on axial motor outcomes

The average coordinates for all electrode contacts were right x (Rx) = -0.228, right y (Ry) = -0.887, right z (Rz) = 1.35, left x (Lx) = -0.471, left y (Ly) = -0.208, and left z (Lz) = 1.23, where lateral (x), anterior (y), and dorsal (z) are defined as positive. Multiple linear regression was next performed to investigate the effect of active contact location (in x-axis, y-axis, and z-axis coordinates) on total axial score (OFF MED-ON STIM), controlling for LED reduction, total motor improvement, and total axial score (OFF MED-OFF STIM) (Table 3).

**Table 3:** Multiple linear regression results using individual axis coordinates as independent variables

	Variable	Slope	Standard error	95% CI (asymptotic)	*P* value	*P* value summary
**Total axial score**	Rx	0.05304	0.1501	-0.2473 to 0.3533	0.7251	ns
	Ry	-0.1248	0.1603	-0.4454 to 0.1958	0.4393	ns
	Rz	0.4070	0.1386	0.1297 to 0.6843	0.0047	**
	Lx	0.03559	0.1548	-0.2741 to 0.3453	0.8190	ns
	Ly	0.06608	0.1727	-0.2793 to 0.4115	0.7033	ns
	Lz	-0.1225	0.1577	-0.4380 to 0.1930	0.4404	ns
	LED reduction	-0.0008477	0.0005099	-0.001868 to 0.0001723	0.1017	ns
	Total motor improvement (OFF MED-OFF STIM minus OFF MED-ON STIM)	-0.07827	0.02156	-0.1214 to -0.03515	0.0006	***
	Total axial score (OFF MED-OFF STIM)	0.7335	0.04484	0.6438 to 0.8232	< 0.0001	****

The Rz coordinate was associated with total axial score (slope = 0.407, *p* = 0.0047), indicating that a 1 unit increase in distance along the right dorsal axis results in a 0.407 increase (worsening) in total axial score. Improvement in total motor score correlated with decreased (improved) total axial score (slope = -0.0783, *p* = 0.0006). LED reduction was not significantly associated with total axial score.

Overall, these data suggest that improvement in total motor score is associated with decreased (improved) total axial score, and the right dorsal axis of STN DBS is associated with increased (worse) total axial score when moving in the ventral to dorsal direction.

### Effect of electrode cluster location on axial motor outcomes

**Fig. 1:**
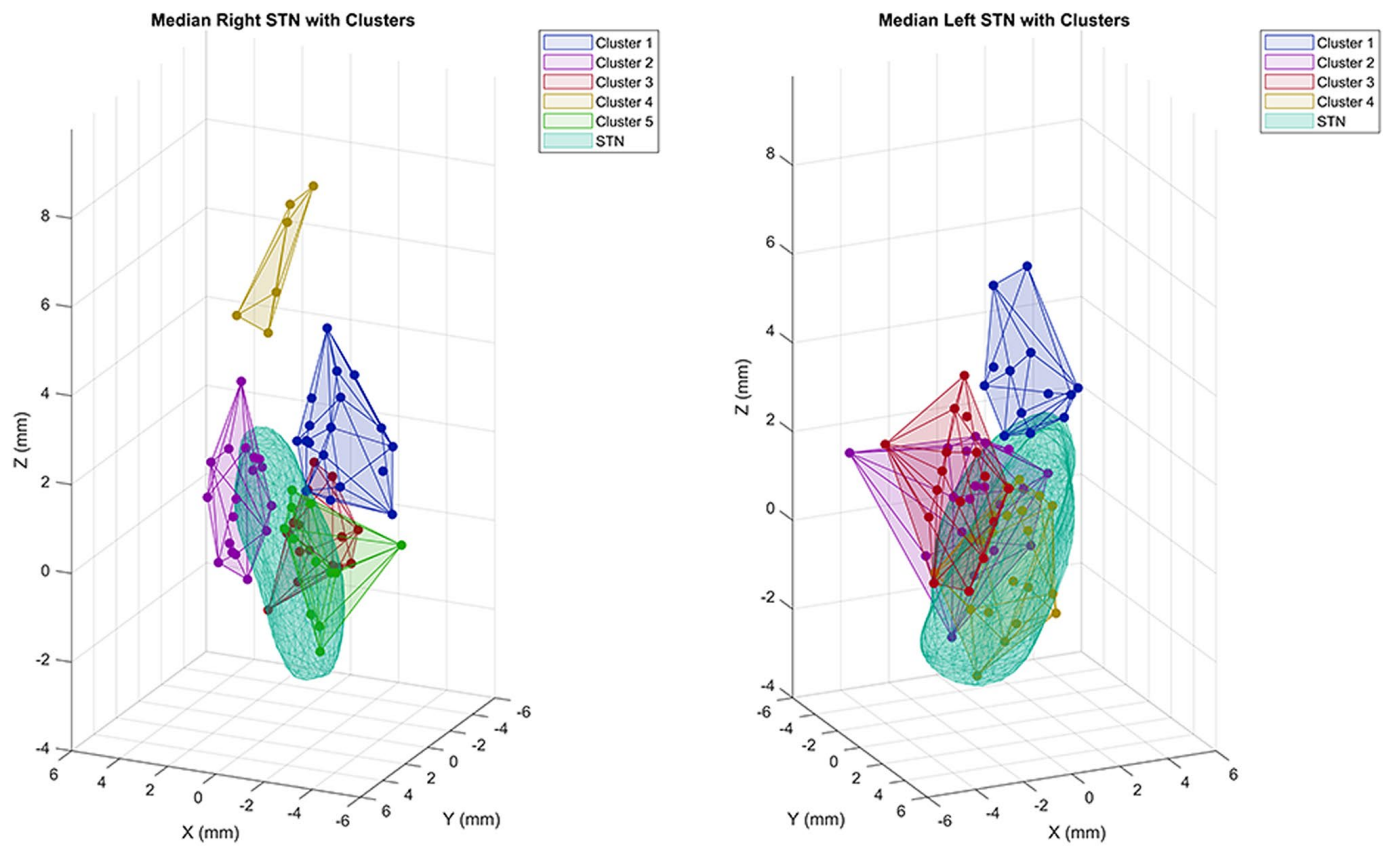
Electrode contact locations by cluster, superimposed onto a representative right STN (figure left) and left STN (figure right). The x-axis represents lateral-medial, y-axis represents anterior-posterior, and z-axis represents dorsal-ventral, where lateral, anterior, and dorsal directions were defined as positive

Since the position of electrode contact along a specific axis is constrained by the lead trajectory, K-means cluster analysis was performed to cluster right and left coordinate data into five and four groups, respectively, based on 3D location (Fig. 1). Final cluster centers are shown in Online Resource 2. Least squares multiple linear regression was then performed to investigate the effect of cluster location on total axial score, controlling for the same variables as the previous regression analysis. Because the dorsal region of the STN has been shown to be associated with appendicular motor improvement and the right z-axis was shown to be associated with total axial score in our regression analysis using individual coordinates, we chose the clusters that were most dorsal on each side as the reference clusters for the following regression analysis. These reference clusters were cluster 4 on the right and cluster 1 on the left.

The results of the regression for total axial score are shown in Table 4. On the right, cluster 5 (R5) was associated with improved total axial score (slope = -2.87, *p* = 0.0154), indicating that the total axial score in this cluster is 2.87 points less (improved) compared to the most dorsal cluster. Cluster 2 on the right (R2) was also associated with improved total axial score (slope = -2.46, *p* = 0.0284) compared with the most dorsal cluster. R5 was located more medial (x = -1.53) and more ventral (z = -0.11) compared with the reference cluster. R2 was located more lateral (x = 1.26) and more ventral (z = 1.17) compared with the reference cluster. Although cluster 3 (R3) was located most ventral (z = -0.39), it was not associated with improved total axial score. R2 and R5 were also associated with improved arising from chair score (slope = -0.749, *p* = 0.018, and slope = -0.729, *p* = 0.0318, respectively). All clusters on the right side were associated with improved postural stability compared with the reference cluster (all *p* < 0.05). On the left, cluster 4 was associated with worsened postural stability (slope = 0.804, *p* = 0.0289). This cluster was located more ventral and medial compared with the reference cluster on that side. On the left, cluster 2 was associated with improved posture compared with the reference cluster (slope = -0.312, *p* = 0.0254). This cluster was located more ventral, medial, and posterior compared with the reference cluster.

**Table 4:** Multiple linear regression results using clusters as independent variables, where right cluster 4 and left cluster 1 are reference levels

	Variable	Slope	Standard error	95% CI (asymptotic)	*P* value	*P* value summary
**Total axial score**	Right cluster[1]	-2.087	1.129	-4.346 to 0.1715	0.0695	ns
	Right cluster[2]	-2.464	1.097	-4.659 to -0.2694	0.0284	*
	Right cluster[3]	-2.153	1.146	-4.445 to 0.1395	0.0652	ns
	Right cluster[5]	-2.871	1.150	-5.173 to -0.5692	0.0154	*
	Left cluster[2]	0.1538	0.8699	-1.587 to 1.895	0.8603	ns
	Left cluster[4]	0.9458	0.8099	-0.6747 to 2.566	0.2476	ns
	Left cluster[3]	1.046	0.7974	-0.5495 to 2.642	0.1946	ns
	LED reduction	-0.001130	0.0004823	-0.002095 to -0.0001649	0.0225	*
	Total motor improvement (OFF-OFF minus OFF-ON)	-0.06898	0.02398	-0.1170 to -0.02101	0.0056	**
	Total axial score (OFF MED-OFF STIM)	0.7066	0.04772	0.6111 to 0.8021	< 0.0001	****

Overall, these results suggest that there may be additional spatial factors contributing to improved axial score on the right in addition to the dorsal-ventral axis. Moreover, the improvement in axial score in R2 and R5 may be partially explained by the improvement in arising from chair score and postural stability. There was an opposite effect on postural stability when associated with a medial, anterior, and ventral location on the right (improved postural stability) vs. the left (worsened postural stability).

## Discussion

### STN DBS improves total axial score

We found that STN DBS improved the total axial score and multiple axial subscores when compared to the OFF MED-OFF STIM condition. There was no improvement in speech which is known to be less likely to respond to DBS (Fasano et al. 2015; Ramirez-Zamora and Ostrem 2018). The combination of STN DBS and medication further improved total axial score and several axial subscores compared to the medication only condition.

Our results are consistent with studies that have reported improvement in some axial symptoms with STN DBS in the short term (Bejjani et al. 2000; Shin et al. 2020). A meta-analysis including 10 studies examining the effects of bilateral STN stimulation at 3, 6, and/or 12 month follow-up showed improvement in postural instability and gait disability (PIGD) assessed using UPDRS items 13–15 and 27–30 compared to preoperative OFF medication scores (Bakker et al. 2004). PIGD was also improved in the ON MED-ON STIM condition vs. the preoperative ON medication condition but at a smaller magnitude. A meta-regression including 9 studies examining bilateral STN stimulation revealed that PIGD (UPDRS items 29–30) improved postoperatively with STN DBS (off medication) compared to the preoperative medication OFF scores, though the improvement gradually decreased over time in contrast to rigidity, bradykinesia, and tremor which remained stably improved (St George et al. 2010).

However, other studies have reported no change or worsening of axial signs with STN DBS. Mei et al. found no difference in the OFF MED-ON STIM axial score (UPDRS items 27–30) at 6 or 36 months compared to baseline (Mei et al. 2020). Zampogna et al. found that axial scores (sum of UPDRS/MDS-UPDRS scores for arising from chair, posture, gait, and postural stability) were similar at 1 year (ON MED-ON STIM) compared with preoperative baseline (ON MED) in 302 patients (Zampogna et al. 2022).

### Electrode contact location and axial motor outcomes

Our study revealed that a more dorsal electrode contact location in the right STN was associated with worse total axial score. When assessing the effect of contact location by cluster, there were two cluster locations on the right that were associated with improved total axial score. These clusters were located more ventral compared to the reference cluster. R2 was located more lateral and R5 was located more medial, suggesting that the lateral-medial axis may be less contributory towards axial score improvement within the ventral STN. Both of these clusters were located anteriorly on the y-axis. In contrast, R3, which was the most ventrally located cluster, was located more posteriorly and was not associated with improved total axial score, suggesting that the anterior-posterior axis may be relevant within the ventral region of the STN.

A more ventral location of potential axial motor benefit is notable since the dorsolateral STN is most often targeted during programming due to the beneficial effect on appendicular symptoms. Moreover, our research group previously used atlas-independent electrophysiologic mapping to identify an optimal locus of stimulation for total motor improvement which was located dorsal, lateral, and posterior to the STN midpoint using part III of the UPDRS/MDS-UPDRS at 6-month follow-up (Conrad et al. 2018). Although R3 was the most ventral cluster on the right and might be expected to be associated with improved axial score, its posterior location may partially explain the lack of association as opposed to R2 and R5 which were both located anterior on the y-axis.

A few studies have investigated the role of stimulation location on axial symptoms with mixed results. In twelve PD patients with stimulation-induced worsening of gait, the ON MED-ON STIM condition resulted in worsened gait (via Stand Walk Sit Test) compared to ON MED-OFF STIM. Most of the contacts responsible for the worsened gait in this study were located dorsal and anterior to the STN (Fleury et al. 2016), similar to our reference clusters. Hilliard et al. found that improvement in gait (via Extended Timed-Get-Up-and-Go test) was observed with more ventral contacts in ten PD patients treated with bilateral STN DBS, whereas tremor was improved with dorsal and anterior contacts (Hilliard et al. 2011). In contrast, Shenai et al. demonstrated that a region of efficacy for improvement of “midline” motor symptoms (UPDRS items 18–19, 27–30) was located in the dorsal STN and caudal zona incerta in 73 patients treated with unilateral DBS at 3-month follow-up compared to baseline (Shenai et al. 2015). McNeely et al. found no difference in gait and balance parameters between unilateral dorsal vs. ventral STN DBS (*N* = 23) (McNeely et al. 2011).

Axial improvement in the ventral region of the STN may be partially explained by the somatotopic organization of the STN. Sasaki et al. retrospectively identified movement-related cells in the STN; upper limb-related cells were more often located in the dorsal, anterior, and lateral regions compared with lower limb-related cells (Sasaki et al. 2019). Moreover, movement-related cells associated with distal joints were more dorsolateral compared with cells associated with proximal joints. Whereas the primary motor cortex projects to the dorsolateral STN, Brodmann area 6 (premotor and supplementary motor cortex) was found to have connections more ventromedial in the STN (rostral area 6) and more ventrolateral in the STN (caudal area 6) in studies of non-human primates (Haynes and Haber 2013). Interestingly, Rodriguez-Rojas suggests a fourth functional region which is located between the sensorimotor and associative regions; this region receives projections from the supplementary motor area (Rodriguez-Rojas et al. 2022). In summary, although the ventral STN is most commonly associated with associative and limbic circuits, it has also been shown to have connections to motor-related circuits, suggesting overlap of functional regions (Emmi et al. 2020).

Importantly, stimulation of the ventromedial STN may result in neuropsychiatric adverse effects such as hypomania (Chopra et al. 2012). This may limit the extent to which the ventral STN may be targeted during DBS programming. Clinicians must also balance the potential re-emergence of tremor, rigidity, etc. if choosing to target a more ventral STN region for axial improvement.

### Asymmetric effects of bilateral stimulation on axial symptoms?

We found that a more ventral location on the right, but not the left, was associated with improved total axial score. Moreover, there was an opposite relationship between a medial, anterior, and ventral cluster location in the right STN (improved postural stability) and left STN (worse postural stability). Although not extensively studied, there are data suggesting potential lateralized effects of stimulation in some patients, particularly on axial symptoms (Lin et al. 2021). In a study of 22 PD patients treated with bilateral STN DBS, 50% were found to have a “dominant STN” based on hierarchical agglomerative cluster analysis. Bilateral or unilateral dominant STN stimulation both improved gait (item 29) and axial score (sum of UDPRS items 18–19, 22, 27–31), whereas unilateral non-dominant STN stimulation did not (Castrioto et al. 2011). These results were supported by another study following the same clustering method in 10 patients with PD; notably, patients with a “dominant STN” had higher baseline, medication-off scores for postural stability and gait (Rizzone et al. 2017). In a randomized, double-blinded, double-crossover trial evaluating 22 PD patients in the ON medication state, reducing left-sided amplitude for ≥ 21 days resulted in improvement in axial score, although significance was not retained after correction for multiple comparisons (Lizarraga et al. 2022).

### Limitations

Our study has several limitations, including the retrospective nature of the analysis. We only looked at electrode location as opposed to calculating a volume of tissue activated (VTA) model. Additionally, our study only looks at short-term follow up of 6 or 12 months; although we demonstrate a significant benefit of STN DBS on most axial motor signs, we cannot comment on sustained responses past these time points. Some studies have shown lack of improvement or worsening of axial signs with STN DBS or STN DBS plus medication at long-term follow-up compared with the respective baseline (Castrioto et al. 2011; Zampogna et al. 2022). Follow up study with longer intervals would be necessary to evaluate long-term effects of STN DBS on axial signs in our cohort. Additionally, we evaluated the motor scores only an hour after turning DBS on or off, which may underestimate the change due to stimulation or withdrawal of stimulation. The 1-hour limit in our study is a practical compromise for our patients since they often do not tolerate being off stimulation for an extended period of time. We were still able to detect a difference in some axial signs after turning DBS on or off for 1 h. It has been shown that 75% of maximal change for axial signs is between 30 and 60 min when turning DBS off, and when turning DBS on, axial signs were significantly decreased after 1 h (Temperli et al. 2003). Other studies have used similar ON/OFF stimulation parameters of 30 min to 1.5 h (Gervais-Bernard et al. 2009; Khoo et al. 2014; Lau et al. 2019). Finally, the axial symptoms in our study were dopa-responsive at baseline, so our results may not be generalizable to patients with axial symptoms fully resistant to dopaminergic medication.

In our regression analysis, there may be a relationship between the electrode contact coordinate variables since the contact locations are constrained by the requirement of lying along the lead trajectory; however, the variance inflation factor (VIF) of each variable is less than 5 in the regression models, suggesting that multicollinearity is unlikely to be a problem. The cluster analysis is also limited by the necessary interpretation of results in reference to one of the other clusters due to the categorical nature of the independent variables in this case. Although the reference cluster on the right is located noticeably more dorsal compared with the reference cluster on the left, it was included in the analysis because of clinical relevance; the active electrode contacts chosen for chronic stimulation based on best clinical effect are often located outside of the STN, just dorsal to the STN or within the caudal zona incerta (Mostofi et al. 2019; Conrad et al. 2018; Kluin et al. 2022).

Differences in results between our study and others may also stem in part from the heterogenous methods of assessing axial improvement. Our study directly compares the follow-up OFF MED-ON STIM condition to the follow-up OFF MED-OFF STIM condition, whereas other studies compare the follow-up OFF MED-ON STIM condition to the baseline (pre-surgery) OFF MED condition. The latter comparison may be subject to confounding variables such as microlesion effect secondary to the surgery or the effect of disease progression, especially at longer follow-up intervals. Additionally, there are many ways to define an axial score in the literature. We used the MDS-UPDRS scale for speech, neck rigidity, arising from chair, gait, freezing of gait, postural stability, and posture, whereas other studies use only some of these subscores or use part II items which are subjective measures of symptoms that are not assessed in specific treatment conditions. Further, some studies use specific tests/scales for gait or balance rather than the UPDRS/MDS-UPDRS.

### Future directions

Although our results suggest that a more ventral electrode contact in the right STN may improve axial signs, prospective studies evaluating the effect of more ventral contacts on axial motor symptoms in other cohorts are warranted. It would also be important to further investigate whether right- vs. left-sided ventral STN stimulation differentially affects axial outcomes in other cohorts. Only high frequency stimulation was used in our study. Some studies have suggested that low frequency stimulation may improve axial signs; moreover, low frequency stimulation in a ventral location may be more optimal for improving axial symptoms in STN DBS (Xie et al. 2017; Khoo et al. 2014). Future studies may consider investigating the effect of active contact location on axial symptoms at different stimulation frequencies.

## Conclusions

Total axial score and several axial subscores were improved with bilateral STN DBS at short-term follow-up. A more ventral electrode contact location in the right STN was associated with improved total axial score, and clusters located both anterior on the y-axis and more ventral compared to the reference cluster were associated with improved total axial score on the right. Our results highlight the efficacy of STN DBS in improving most axial motor signs in PD patients and support the role of electrode contact location on differential axial motor response. Further research is warranted to determine whether stimulation of the ventral STN is the optimal location for improving axial signs in individual patients.

## Electronic supplementary material

Below is the link to the electronic supplementary material.

Supplementary Material 1

Supplementary Material 2

## Data Availability

The data supporting the results of this study are available from the corresponding author upon reasonable request.
