# Supplementary material for: Ventral STN stimulation is associated with improved axial motor outcomes in Parkinson’s disease
Source: J Neural Transm (Vienna). 2025 Apr 24;132(7):1031–40. doi: 10.1007/s00702-025-02931-1 (PMC12209391; doi:10.1007/s00702-025-02931-1)
Supplement: Supplementary file 2 — Supplementary Material 2 [file 702_2025_2931_MOESM2_ESM.docx]

**Online Resource 2.** Final cluster centers from K-means cluster analysis

|  | **Cluster 1** | **Cluster 2** | **Cluster 3** | **Cluster 4** | **Cluster 5** |
| --- | --- | --- | --- | --- | --- |
| **Rx** | -1.35 | 1.31 | 0.21 | -0.04 | -1.53 |
| **Ry** | -1.94 | 0.96 | -3.16 | 0.50 | 0.25 |
| **Rz** | 2.26 | 1.20 | -0.39 | 6.59 | -0.11 |
|  | ***Cluster 1*** | ***Cluster 2*** | ***Cluster 3*** | ***Cluster 4*** |  |
| **Lx** | 0.76 | 0.00 | -2.52 | -0.12 |  |
| **Ly** | 0.43 | -2.62 | 1.32 | 0.53 |  |
| **Lz** | 3.84 | 0.51 | 1.87 | -0.19 |  |
